# Functional Significance of SRJ Domain Mutations in *CITED2*

**DOI:** 10.1371/journal.pone.0046256

**Published:** 2012-10-17

**Authors:** Chiann-mun Chen, Jamie Bentham, Catherine Cosgrove, Jose Braganca, Ana Cuenda, Simon D. Bamforth, Jürgen E. Schneider, Hugh Watkins, Bernard Keavney, Benjamin Davies, Shoumo Bhattacharya

**Affiliations:** 1 Department of Cardiovascular Medicine, Wellcome Trust Centre for Human Genetics, University of Oxford, Oxford, United Kingdom; 2 Wellcome Trust Centre for Human Genetics, University of Oxford, Oxford, United Kingdom; 3 Institute of Genetic Medicine, Newcastle University, Newcastle, United Kingdom; 4 Centro Nacional de Biotecnología, CSIC, Madrid, Spain; IRCCS-Policlinico San Donato, Italy

## Abstract

CITED2 is a transcriptional co-activator with 3 conserved domains shared with other CITED family members and a unique Serine-Glycine Rich Junction (SRJ) that is highly conserved in placental mammals. Loss of *Cited2* in mice results in cardiac and aortic arch malformations, adrenal agenesis, neural tube and placental defects, and partially penetrant defects in left-right patterning. By screening 1126 sporadic congenital heart disease (CHD) cases and 1227 controls, we identified 19 variants, including 5 unique non-synonymous sequence variations (N62S, R92G, T166N, G180-A187del and A187T) in patients. Many of the CHD-specific variants identified in this and previous studies cluster in the SRJ domain. Transient transfection experiments show that T166N mutation impairs TFAP2 co-activation function and ES cell proliferation. We find that CITED2 is phosphorylated by MAPK1 *in vitro* at T166, and that MAPK1 activation enhances the coactivation function of CITED2 but not of CITED2-T166N. In order to investigate the functional significance *in vivo*, we generated a T166N mutation of mouse *Cited2*. We also used PhiC31 integrase-mediated cassette exchange to generate a *Cited2* knock-in allele replacing the mouse *Cited2* coding sequence with human *CITED2* and with a mutant form deleting the entire SRJ domain. Mouse embryos expressing only CITED2-T166N or CITED2-SRJ-deleted alleles surprisingly show no morphological abnormalities, and mice are viable and fertile. These results indicate that the SRJ domain is dispensable for these functions of CITED2 in mice and that mutations clustering in the SRJ region are unlikely to be the sole cause of the malformations observed in patients with sporadic CHD. Our results also suggest that coding sequence mutations observed in case-control studies need validation using *in vivo* models and that predictions based on structural conservation and *in vitro* functional assays, or even *in vivo* global loss of function models, may be insufficient.

## Introduction

Congenital heart disease (CHD) is one of the major causes of childhood morbidity and mortality in the West. The incidence of CHD in live-born infants ranges from 0.4 to 1.2% [1], [2], and increases in first-degree relatives to 2–5% [2], suggesting a role for genetic or environmental variations which may contribute to disease risk. Chromosomal and Mendelian syndromes account for approximately 20% (11.9% and 7.4% respectively) of CHD cases [3], [4]. The genetic architecture underlying the remaining 80% of “sporadic” CHD remains elusive and cannot be addressed by standard family based linkage studies. However, genetic variants have been shown to be associated with sporadic, non-Mendelian/non-chromosomal CHD as non-synonymous disease-associated mutations have previously been found in case-control studies [5].

CITED2 is a CREBBP/EP300-interacting protein that is present in all vertebrates. It is highly conserved in placental mammals, with 95% identity between human and mouse. It has three regions (CR1-3) that are conserved in other CITED family members, and also an unusual Serine-glycine Rich Junction (SRJ, residues 161–199), which is unique to CITED2 [6]–[10]. The function of CR2 (residues 215–270) is to bind the CH1 domain of CREBBP and EP300 transcriptional co-activators, and *in vitro* studies indicate that it is necessary for all known biological activities of CITED2 [10]–[13]. CITED2 competitively inhibits hypoxia-activated gene transcription by blocking the interaction between CREBBP/EP300 and HIF-1A [10]. CITED2 also functions as a transcriptional co-activator, by recruiting CREBBP/EP300 to chromatin via the DNA-binding transcription factor AP2 (TFAP2) [11], [14], [15]. The functions of CR1, CR3 and the SRJ domain are not known. The SRJ domain has been hypothesized to be a mutational hotspot as variants clustering in this region have previously been reported in patients with CHD [16], [17].

*Cited2* is essential for normal mouse development. Mice lacking *Cited2* die *in utero* with cardiac and aortic arch malformations, adrenal gland agenesis, small cranial and dorsal root ganglia, exencephaly, and neural crest and left-right patterning defects [11], [15], [18]–[22]. The cardiac malformations in mice lacking *Cited2* are diverse and include atrial and ventricular septal defects, double outlet right ventricle, common arterial trunk, tetralogy of Fallot, transposition of the great arteries, and interrupted and aberrant aortic arches.

In this study, we have investigated the involvement of *CITED2* in CHD by direct sequencing of a cohort of CHD patients and controls and confirmed the clustering of non-synonymous mutations to the SRJ domain. *In vitro* experiments indicated that a specific residue in the SRJ domain (T166) was a functional target of MAPK1, and was necessary for TFAP2 co-activation. We used gene-targeting technologies in the mouse to functionally assess the contribution of T166 and the SRJ domain, as a whole, to disease. Mouse embryos expressing only CITED2-T166N or CITED2 SRJ-deleted alleles surprisingly showed no structural abnormalities by magnetic resonance imaging, and mice were viable and fertile. These results suggest that the SRJ domain is dispensable for CITED2 function in mice. Thus, point mutations and deletions clustering in the SRJ region are unlikely to be the sole cause of the malformations observed in patients with sporadic CHD, and may require additional factors to cause disease.

## Results

### Rare variants are infrequently found in *CITED2* in CHD and cluster in the SRJ domain

We sequenced the entire *CITED2* open reading frame in 1126 CHD cases and 1227 ethnically matched controls (Table 1 and Table S1). Nineteen sequence variants were identified. Of the non-synonymous variants found, five (T166N, R92G, N62S, A187T and G180-A187del) were unique to cases, while two (P36R, Q40H) were present only in controls. Three non-synonymous variants (H39del, H160L, G194-G195del) were present in both cases and controls. Three of the five non-synonymous variants found only in cases were inherited from unaffected parents (N62S, T166N and G180-A187del). Although there was an excess of non-synonymous variants that were unique to cases, this was not statistically significant using a 2-tailed Fisher's exact test. H39del was found in 10 cases and 1 control; however, we were unable to confirm this ratio in a replicate cohort (9/566 in cases vs. 43/2394 in controls, p = n.s., Text S1).

**Table 1:** Synonymous and non-synonymous mutations identified through direct sequencing of 1126 cases with CHD and 1227 controls.

Variation	Protein	Cases	Controls	Diagnosis
**Non-Synonymous, Unique**				
c.559G>A	p.Ala187Thr	1	0	PS
c.497C>A	p.Thr166Asn	1	0	TGA
c.274A>G	p.Arg92Gly	1	0	ASD
c.185A>G	p.Asn62Ser	1	0	TOF
c.538-561del	p.Gly180_Ala187del	1	0	AVSD
c.107C>G	p.Pro36Arg	0	1	Control
c.120G>C	p.Gln40His	0	1	Control
**Synonymous, Unique**				
c.762T>C	p.Asp254Asp	1	0	Ebstein,VSD
c.612C>T	p.Ser204Ser	1	0	TGA
c.471C>T	p.Asn157Asn	1	0	ASD
c.381C>T	p.His127His	1	0	TOF
c.276G>A	p.Arg92Arg	1	0	VSD
c.117C>T	p.His39His	0	1	Control
c.120G>A	p.Gln40Gln	0	1	Control
**Both**				
c.115-117delCAC	p.His39del	10	1	
c.479A>T	p.His160Leu	11	9	
c.582C>T	p.Gly194Gly	7	13	
c.21C>A	p.Ala7Ala	306	336	
c.580-585delGGCGGC	p.Gly194_Gly195del	6	10	

5 unique non-synonymous variants are observed in the cases and 2 in the control group. Abbreviations: PS, pulmonic stenosis; TGA, transposition of great arteries; ASD, atrial septal defect; TOF, Tetralogy of Fallot; AVSD, atrioventricular septal defect; VSD, ventricular septal defect.

**Figure 1:**
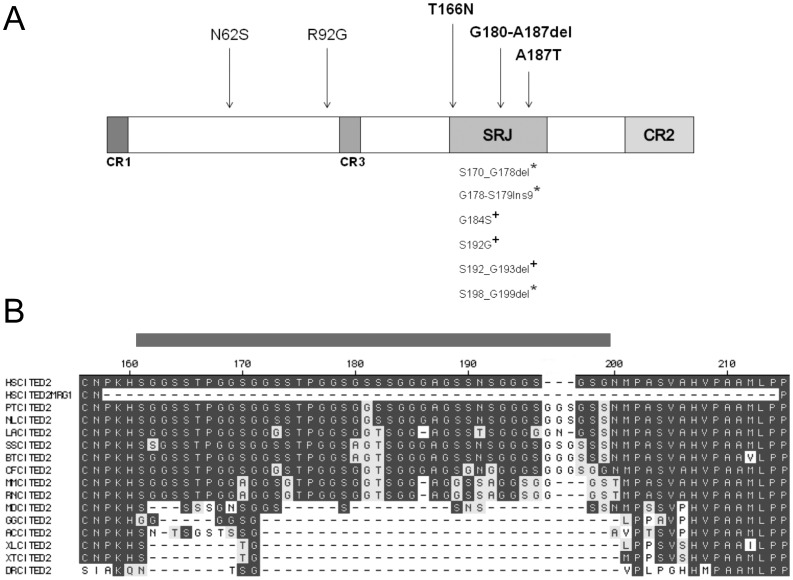
Structure of CITED2. (A) Schematic diagram of human CITED2 showing conserved regions (CR) 1–3 and the serine-glycine rich junction (SRJ). Also indicated are the locations of CITED2 variants. All unique non-synonymous variants found in this study are shown above the figure with variants found in the SRJ highlighted. Unique non-synonymous variants found by Sperling et al., 2005 (*) and Yang et al., 2010 (^+^) are shown below. SRJ comprises AA 161 to 199. (B) The CITED2 peptide sequence is shown for *Homo sapiens* (Human, HSCITED2, AF129290), CITED2-MRG1 (Human, HSCITED2 MRG1 isoform lacking the entire SRJ), *Pan troglodytes* (Chimapanzee, PTCITED2), *Nomascus leucogenys* (Gibbon, NLCITED2), *Loxodonta africana* (Elephant, LACITED2), *Sus scrofa* (Pig, SSCITED2), *Bos taurus*, (Cow, BTCITED2), *Canis familiaris* (Dog, CFCITED2), *Mus musculus* (Mouse, MMCITED2), *Rattus norvegicus* (Rat, RNCITED2), *Monodelphis domestica* (Opossum, MDCITED2), *Gallus gallus* (Chicken, GGCITED2), *Anolis carolinensis* (Anole lizard, ACCITED2), *Xenopus laevis* (African clawed frog, XLCITED2), *Xenopus tropicalis* (Western clawed frog, XTCITED2), and *Danio rerio* (Zebrafish, DRCITED2). Sequence alignments taken from the latest genome builds from EBI Ensembl. SRJ domain spans region marked with grey bar above corresponding to AA161 to 199 in the human protein sequence.

Three of the five non-synonymous variants identified uniquely in cases in this study, three of six in the study by Sperling et al. [16] and four of four in the study by Yang et al. [17], lie within the SRJ region of *CITED2* (Fig. 1A). The SRJ domain is highly conserved in placental mammals. It is, however, substantially abbreviated in marsupials and is absent in other vertebrates (Fig. 1B). Using the RONN program [23], we find that the SRJ domain is predicted to have a highly disordered secondary structure. However, none of the SRJ domain mutations listed above affected the disorder plot to any significant extent (Fig. S1). A number of functions have been indicated for intrinsically disordered regions, including molecular recognition, ligand binding, protein, DNA and RNA interactions, and as flexible linkers between domains [24].

### CITED2 T166N mutation ablates TFAP2 coactivation function in vitro

**Figure 2:**
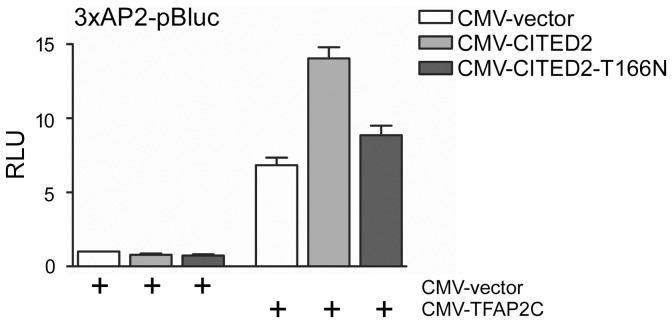
CITED2 mutation and its functional consequences. Hep3B cells were transiently transfected with the 3xAP2-luciferase reporter and the indicated plasmids and with CMV-lacZ. Results (mean±SEM, three independent experiments) are presented as relative luciferase units (RLU), corrected for β-galactosidase activity. The control transfection value (at the extreme left) in each case (with CMV-vector) is set at 1.

Using the program NetPhos, we found that T166 is predicted to be a phosphorylation site for proline directed kinases [25], [26]. The T166N mutation is predicted to abolish this putative phosphorylation site. To assess the functional significance of the T166N variant we examined its ability to co-activate a TFAP2 isoform. We performed transient transfection assays in Hep3B cells using a TFAP2 luciferase reporter containing three copies of the human metallothionein IIa TFAP2-binding site [11], [14]. As reported previously [11], [14], transfection of CITED2 alone did not affect reporter activity, and co-transfection of TFAP2C and CITED2, enhanced reporter activity 2 fold over that achieved with TFAP2C alone. In comparison, CITED2-T166N was severely defective for TFAP2C co-activation (Fig. 2).

Another molecular function of CITED2 is to represses HIF1 transactivation by interfering with the interaction between the carboxy-terminus of HIF1A, and EP300/CREBBP [10], [27], [28]. We tested the ability of CITED2 to repress the trans-activation of a fusion protein that contains the yeast GAL4 DNA-binding domain fused to the carboxy-terminus transactivation domain of HIF1A (residues 723–826, GAL4-HIF1A [10]). This is a weak transactivator under normoxic conditions but is strongly activated by either hypoxia or the iron chelator desferrioxamine (DFO) [10]. We found that T166N was identical to wild-type CITED2 in repressing GAL4-HIF1A transactivation. We also found that CITED2-T166N is expressed at wild-type levels, binds EP300 and TFAP2 efficiently *in vitro*, and is localised to the nucleus thus excluding these factors as possible mechanisms for impaired function (Fig. S2).

### CITED2 is phosphorylated by MAPK1 at S85, T166 and T175

**Figure 3:**
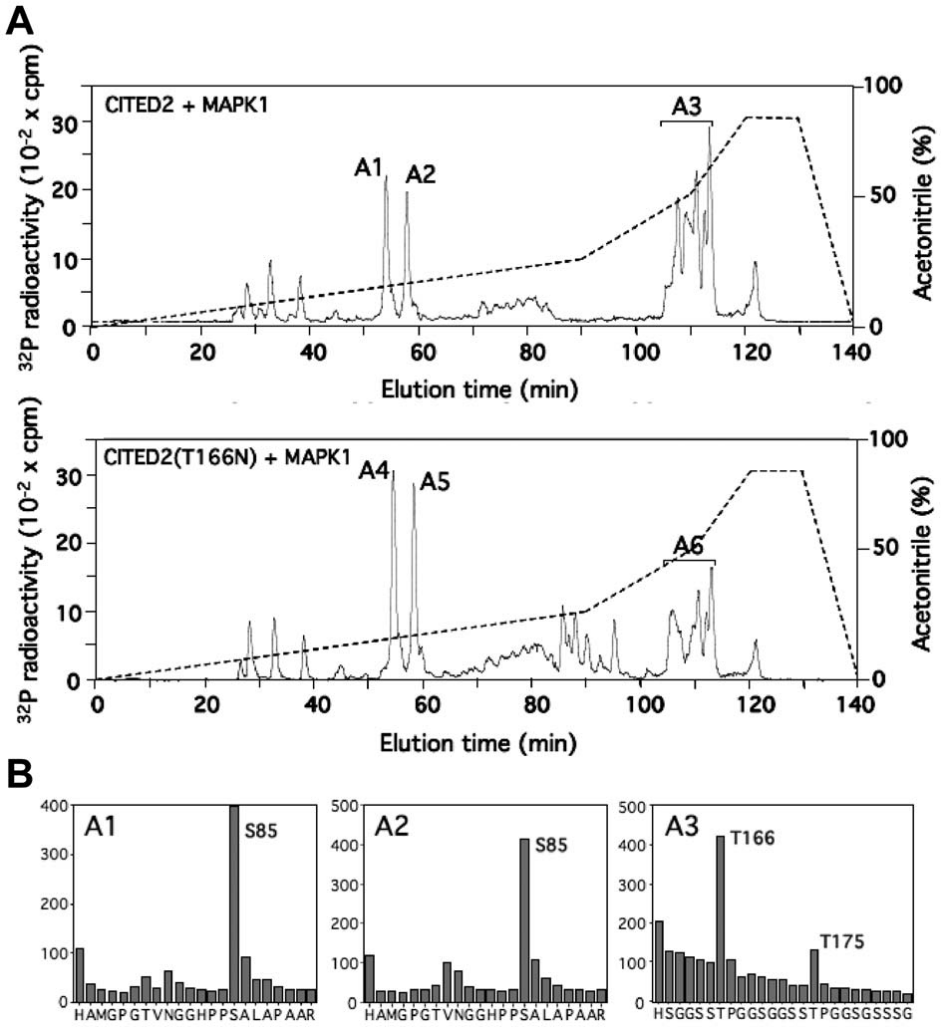
Identification of residues in CITED2 wild type or CITED2T166N phosphorylated by ERK2 (MAPK1). (A) GST-CITED2 wild type or mutant GST-CITED2T166N were incubated with Mg [^32^P]ATP in the presence of ERK2 and subjected to SDS-PAGE. The phosphorylated CITED2 was excised from the gel, digested with trypsin and the peptide separated by chromatography on a Vydac C18 column. The column was developed with an acetonitrile gradient (broken line) and ^32^P-radioactivity is shown (full line). The phosphopeptides A1 to A6 are indicated. (B) The major peaks (A1 to A6) of ^32^P-radioactivity were analysed by MALDI-TOF, MALDI TOF-TOF, Edman degradation and phosphoamino acid analysis as described in Materials and Methods and the data for A1–A3 is shown. The sequence inferred from this data is shown underneath each figure.

We examined the phosphorylation of CITED2 by MAPK1 (ERK2, Fig. 3). Recombinant human CITED2 was phosphorylated *in vitro* by active MAPK1. The phosphorylated CITED2 was then digested with trypsin and the resulting peptides were chromatographed on a Vydac C18 column. Three major peaks of ^32^P radioactivity, termed A1 to A3, were observed. Both peaks A1 and A2 contained a peptide corresponding to residues 71–92 of CITED2 phosphorylated at Ser85. Interestingly, CITED2-Ser85 is not followed by a proline residue. Although all MAP Kinases phosphorylate preferentially Ser or Thr followed by Pro, non-canonical phosphorylation sites, such as the one found in CITED2, have previously been shown [29]–[32]. No mass spectrometry data was obtainable for the A3 group of peaks but phosphoaminoacid analysis indicated that only phosphoThreonine was present (data not shown). In addition, Edman degradation showed the presence of ^32^P at residues 7 and 16. Only one tryptic peptide occurs in CITED2 that is consistent with tryptic cleavage at Lys/Arg followed by Threonine residues at positions 7 and 16 and it consists of residues 160–239, (k)hsggsstpggsggsstpggsgsssgggagssnsgggsgsgnmpasvahvpaamlppnvidtdfideevlmslviemgldr. Varying oxidation states of the 4 Methionine residues in this sequence could account for its complexity on the HPLC elution profile. Taken together, it suggests that peak A3 is a mixture of peaks of ^32^P radioactivity containing a diphosphopeptide corresponding to residues 160–239 phosphorylated at Thr166 and Thr175 (three minor peaks of radioactivity were also observed eluting at 25–40 min, but the identity of the phosphorylated residue(s) eluting in them could not be determined). Phosphorylation of the mutant CITED2-T166N by MAPK1 was also analyzed by chromatography on a Vydac C18 column after tryptic digestion. The same three major (called A4 to A6 in the Fig. 3) and minor peptides phosphorylated on wild type CITED2 were phosphorylated in CITED2-T166N, however, the radioactivity of peak A3 was significantly reduced in the mutant and some other minor peaks appeared (85–95 min on the elution profile). Peaks A4 and A5 contained the peptide (residues 71–92) of CITED2 phosphorylated at Ser85. Peak A6 contained a peptide with ^32^P radioactivity released at cycle 16 and containing phospho-Thr (phosphoaminoacid analysis and Edman degradation, data not shown). On the basis of its similar HPLC elution profile to peak A3 of wild type CITED2, this is most likely the peptide containing residues 160–239 phosphorylated at Thr175. The diphosphopeptide with phosphoThr166 was not found due to the T166N mutation.

### CITED2 coactivation is enhanced by activation of MAPK1 and requires T166

**Figure 4:**
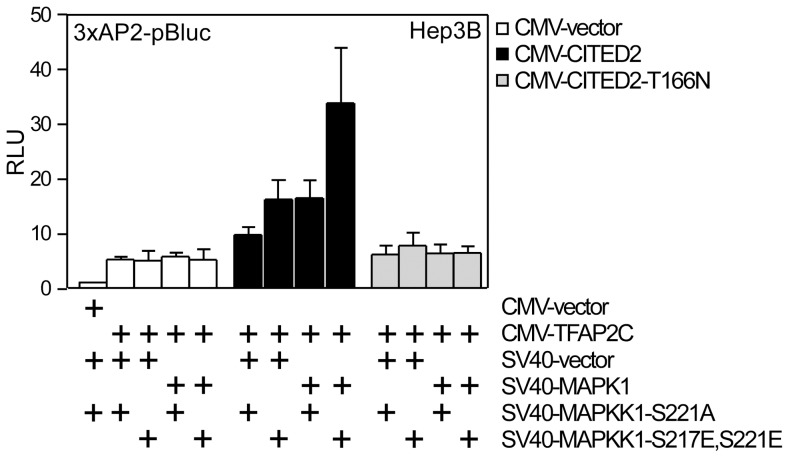
MAPK signalling to CITED2 enhances its co-activation function. Hep3B cells were transiently transfected with the 3xAP2-luciferase reporter, with CMV-lacZ, CITED2-expressing plasmid or the control vector and plasmids expressing MAPK1 or the control vector and with plasmids expressing a defective MAPKK1 (SV40-MAPKK1-S221A) or a constitutively active MAPKK1 (SV40-MAPKK1-S217ES221E). Results are presented as relative luciferase units corrected for lacZ activity. The control transfection value (at the extreme left) in each case (with CMV-vector) is set at 1.

The above results suggested that CITED2 phosphorylation by MAPK1 at T166 may be required for its coactivation function. To test this, we performed coactivation studies in Hep3B cells. Here, we co-transfected a TFAP2 reporter plasmid, and plasmids encoding CITED2, CITED2T166N, MAPK1, and variants of the MAPK1-activating kinase, MAPKK1S221A (a constitutively inactive mutant) or MAPKK1-S217ES221E (a constitutively active mutant). No significant effect of MAPK1 and MAPKK1 plasmids was observed on activity of the reporter in the absence of TFAP2 (not shown) (Fig. 4). However a major effect (3-fold over CITED2 alone) was observed on TFAP2C coactivation by CITED2 in the presence of MAPK1+MAPKK1-S217ES221E. In comparison to TFAP2C without CITED2, this represents a ∼6 fold activation of the reporter (Fig. 4). Co-expression of MAPK1 or MAPKK1 did not affect the expression of CITED2 (Fig. S3). CITED2-T166N did not respond to MAPK1 activation. These results indicate that activated MAPK1 enhances co-activation function of CITED2 (Fig. 4). The effect is specific to TFAP2C in these experiments, and also requires the T166 residue. Taken together these results support the idea that T166 is the target of a proline-directed kinase, and its phosphorylation enhances CITED2 coactivation function.

### T166 is essential for maintaining ES proliferation in the absence of Leukemia Inhibitory Factor (LIF)

**Figure 5:**
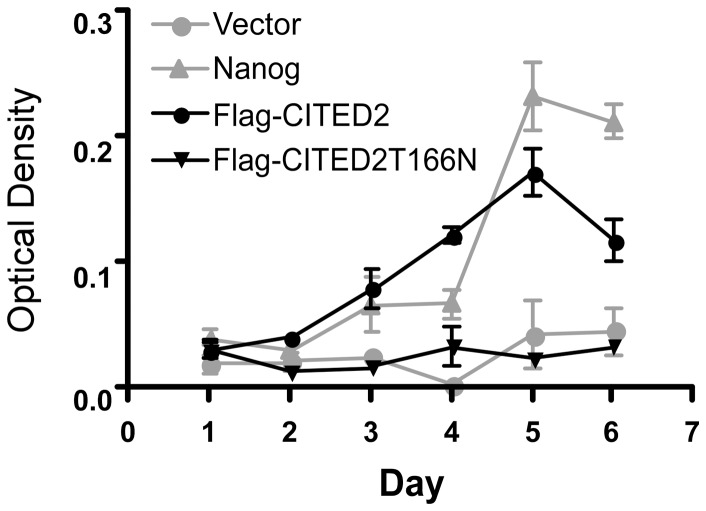
ES cell proliferation rescue by wild-type CITED2 and CITED2-T166N in the absence of leukemia inhibitory factor (LIF). E14/T ES cells transfected by electroporation with the indicated plasmids were selected in puromycin. Surviving cells were plated in quadruplicate into 12-well plates in medium containing puromycin, but in the absence of LIF. The cells were fixed at the indicated time points using 10% formalin. The relative number of ES cells was determined by staining with 0.1% crystal violet, extracting cell-associated dye using 10% acetic acid, and measuring absorbance at A590. Results are presented as mean±SEM. Results represent a single experiment. Similar results were obtained from three independent experiments.

We next sought to determine if T166 is necessary for the ability of CITED2 to maintain mouse embryonic stem (ES) cell proliferation. ES cells can proliferate indefinitely in culture, and are pluripotent [33]. In mouse ES cells this requires the growth factors LIF and BMP [34] and is correlated with the expression of alkaline phosphatase [35]. Gain-of-function experiments have shown that *Nanog* and *Cited2* can bypass the requirement for LIF in mouse ES cells [35]–[37]. We used a previously described episomal expression system in ES cells for these experiments [36]. We transfected plasmids bearing *Nanog*, *CITED2*, *CITED2-T166N*, and empty vector controls in E14/T ES cells. Following puromycin selection, cells were plated in the absence of LIF, and numbers estimated using crystal violet staining at daily intervals thereafter. These experiments showed that both *Nanog* and *CITED2* maintained the proliferation of ES cells in the absence of LIF, whereas empty vector did not. *CITED2-T166N* had a markedly reduced ability to maintain ES cell proliferation in the absence of LIF suggesting that T166 is essential for maintaining ES proliferation in the absence of LIF (Fig. 5).

### Generation of mice harboring the non-synonymous T166N variant within the *Cited2* SRJ domain

**Figure 6:**
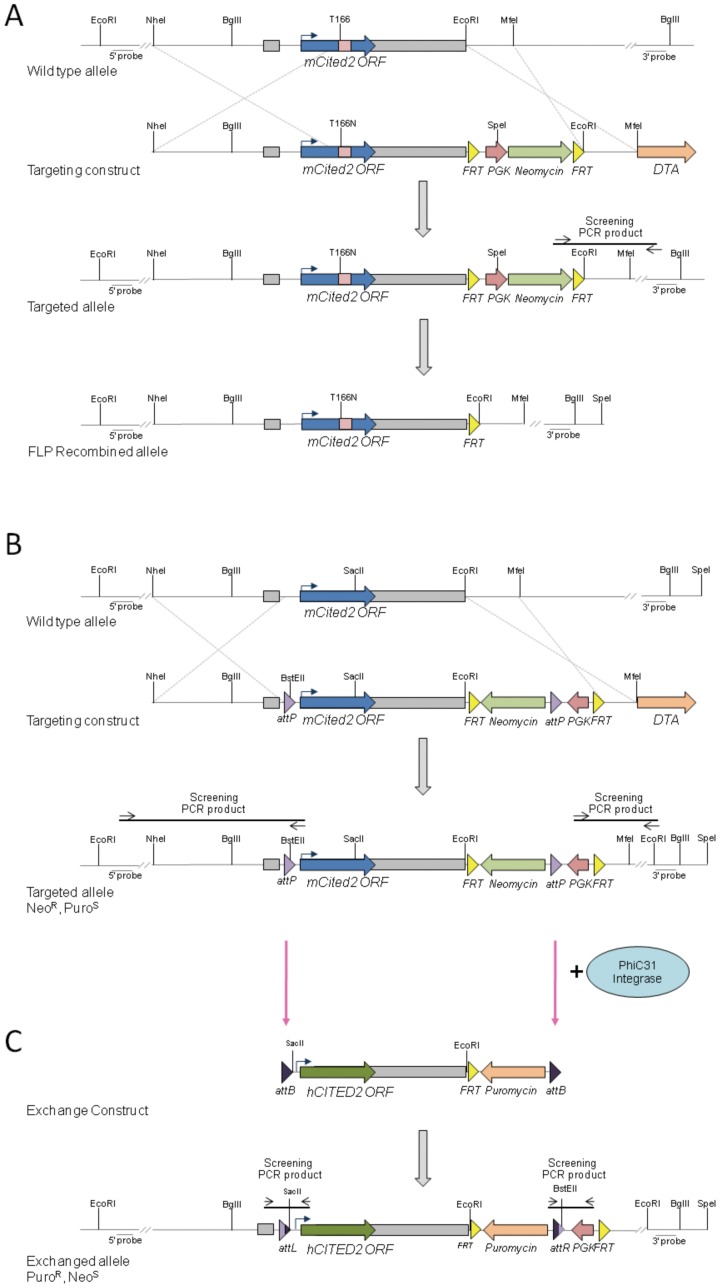
Generation of *Cited2* alleles. (A) Targeting strategy for the generation of *Cited2 ^T166N^* allele by homologous recombination. The T to N change was introduced by site directed mutagenesis into the orthologous residue in the mouse sequence. The structure of the wildtype *Cited2* allele, targeting vector, targeted allele, and its structure after FLP mediated recombination are shown. The open reading frame (ORF, blue arrow) is entirely contained within exon 2 (exons indicated by grey rectangles). The targeting vector has an *frt-PGK-NeoR-frt* selection cassette downstream of exon 2, followed by a DTA (diphtheria toxin) cassette. (B) Targeting strategy for the generation of the *Cited2^attP^* ES cell line. A 5′ *attP* site was introduced into the first intron, upstream of the ATG and a 3′ *attP* site downstream of the stop codon and exon 2 as part of the neomycin selection cassette, in between the PGK promoter and the neomycin coding region. A DTA cassette was also introduced downstream of the 3′ homology arm for negative selection. (C) The human CITED2^MRG1^ isoform (not shown) and the human full length CITED2 were targeted into the *Cited2* locus via PhiC31 integrase mediated cassette exchange. The exchange event occurs between the *attP* and *attB* sites giving rise to *attL* and *attR* sites. Successful exchange replaces the mouse *Cited2* ORF with that of either the MRG1 isoform of *CITED2* or the full length human *CITED2* and brings the puromycin resistance gene under control of the PGK promoter.

**Figure 7:**
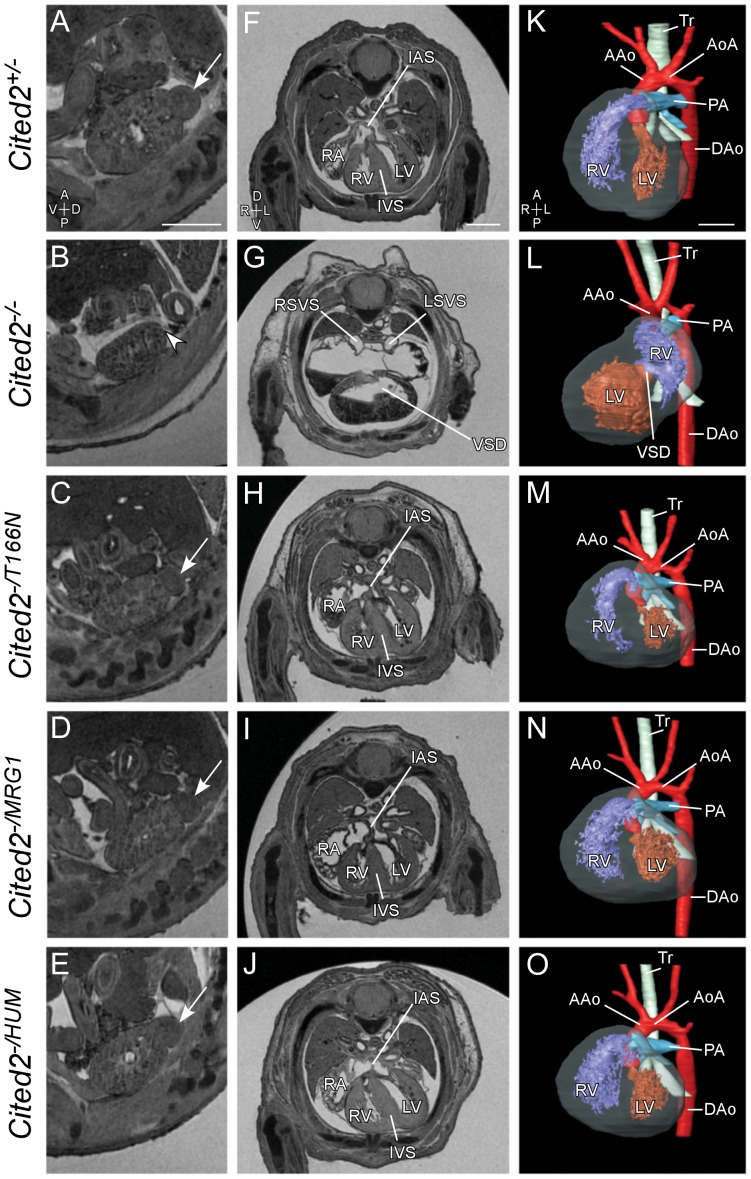
Phenotypic analysis of mouse embryos expressing CITED2 variants. MRI analysis of embryos 15.5 days post coitum (dpc). Genotypes are indicated as shown. Sagittal sections through the left kidney (A–E) are shown to indicate the left adrenal gland where present (arrows), and absent (arrowhead). Transverse sections through the thorax (F–J) and 3D reconstructions (K–O) are shown to demonstrate cardiac anatomy. Loss of *Cited2* leads to adrenal agenesis (B, arrowhead), right atrial isomerism, ventricular septal defect (VSD) and common atrium (G), and abnormal ventricular topology (L). Embryos expressing only the T166N variant, the MRG1 isoform, or full length human CITED2 have normal adrenal glands and hearts. RA, Right Atria; RV, Right Ventricle; LV, Left Ventricle; IVS, Interventricular Septum; IAS, Intra-atrial Septum; AAo, Aorta; AoA, Aortic Arch; Tr, Trachea; DAo, Dorsal Aorta; LSVS and RSVS, Left and Right Systemic Venous Sinus. Axis: A, Anterior; P, Posterior; V, Ventral; D, Dorsal; L, Left; R, Right. Scale bars: 0.5 mm.

In order to investigate the functional significance of the T166N variant *in vivo*, the orthologous amino acid change was introduced into the mouse *Cited2* locus using homologous recombination in ES cells (Fig. 6A). *Cited2 ^+/T166N^* embryos (n = 9) and *Cited2 ^T166N/T166N^* embryos (n = 2) at 15.5 days post coitum (dpc) analyzed by magnetic resonance imaging (MRI) were found to be anatomically normal, showing no dominant effect. In addition, they were viable and fertile. One possibility in the human situation is that the wild-type allele may not sufficiently compensate for lack of activity of the mutant allele. To determine if a single allele of *Cited2 ^T166N^* would support normal embryonic development, we studied *Cited2 ^−/T166N^* mouse embryos. *Cited2 ^−/T166N^* mice were generated by crossing *Cited2 ^+/T166N^* to *Cited2 ^+/−^*. 31 out of 31 *Cited2 ^−/T166N^* mouse embryos at 15.5 days post coitum (dpc) analyzed by magnetic resonance imaging (MRI) were found to be anatomically normal. They revealed none of the structural developmental anomalies associated with the loss of *Cited2* and had normal sized adrenal glands (Fig. 7C, H and M, Fig. S4). Furthermore, *Cited2 ^−/T166N^* mice were born at the expected Mendelian ratios (6/20, Table 2A, *X^2^(3)* = 0.4; p = n.s.) and were viable and fertile.

**Table 2:** Mice expressing a single copy of the *Cited2 ^T166N^* (A) or *Cited2 ^MRG1^* (B) allele are found at the expected Mendelian ratios at weaning.

	Expected	Observed
***Cited2^+/−^ ♂ X Cited2^+/T166N^♀***		
*Cited2^+/T166N^*	5	5
*Cited2^−/T166N^*	5	6
*Cited2^+/+^*	5	5
*Cited2^+/−^*	5	4
***Cited2^+/−^ ♂ X Cited2^+/MRG1^ ♀***		
*Cited2^+/MRG1^*	10.25	10
*Cited2^−/MRG1^*	10.25	12
*Cited2^+/+^*	10.25	10
*Cited2^+/−^*	10.25	9

Mice were genotyped at 3 weeks of age. All genotypes are present at the expected Mendelian ratios.

### Development of a system to efficiently introduce human *CITED2* variants into mouse *Cited2*

In order to introduce human *CITED2* variants into the mouse *Cited2* gene at high efficiency, we adapted a Recombinase Mediated Cassette Exchange (RMCE) system that uses PhiC31 integrase [38], [39]. Using homologous recombination, the *Cited2* gene was targeted in C57BL/6N mouse ES cells so that exon 2 (which contains the entire open reading frame, ORF) became flanked with *attP* sites, resulting in the generation of ES cell line, *Cited2 ^attP^* (Fig. 6B). Using this ES cell line, the *Cited2* ORF can be manipulated and exchanged with sequences introduced using the exchange plasmids harboring analogously positioned *attB* sites. Co-transfection with PhiC31 integrase results in their stable integration at high efficiency within the *Cited2* gene (data not shown).

### Generation of mice lacking the CITED2 SRJ domain

As variants found in patients tend to cluster in the SRJ domain, and having found that a non-synonymous mutation within the SRJ domain was compatible with normal embryonic development, we sought to understand the function of the SRJ domain as a whole by assessing the effect of replacing the mouse *Cited2* ORF with that of the human *CITED2* MRG1 isoform [6], [7]. This isoform lacks residues 158–214 (i.e. the SRJ region plus 18 flanking amino acids) and is a rare variant of CITED2 (ENSP00000376126, Fig. 1B) which likely arises from non-canonical splice sites within exon 2 [10]. In order to achieve this, the endogenous mouse *Cited2* ORF in *Cited2 ^attP^* ES cells was replaced using RMCE with the *CITED2 ^MRG1^* ORF (referred to as *Cited2 ^MRG1^*) and its corresponding control, full-length human *CITED2* ORF (referred to as *Cited2 ^HUM^*, Fig. 6C).

### The SRJ domain of CITED2 is dispensable for function

**Figure 8:**
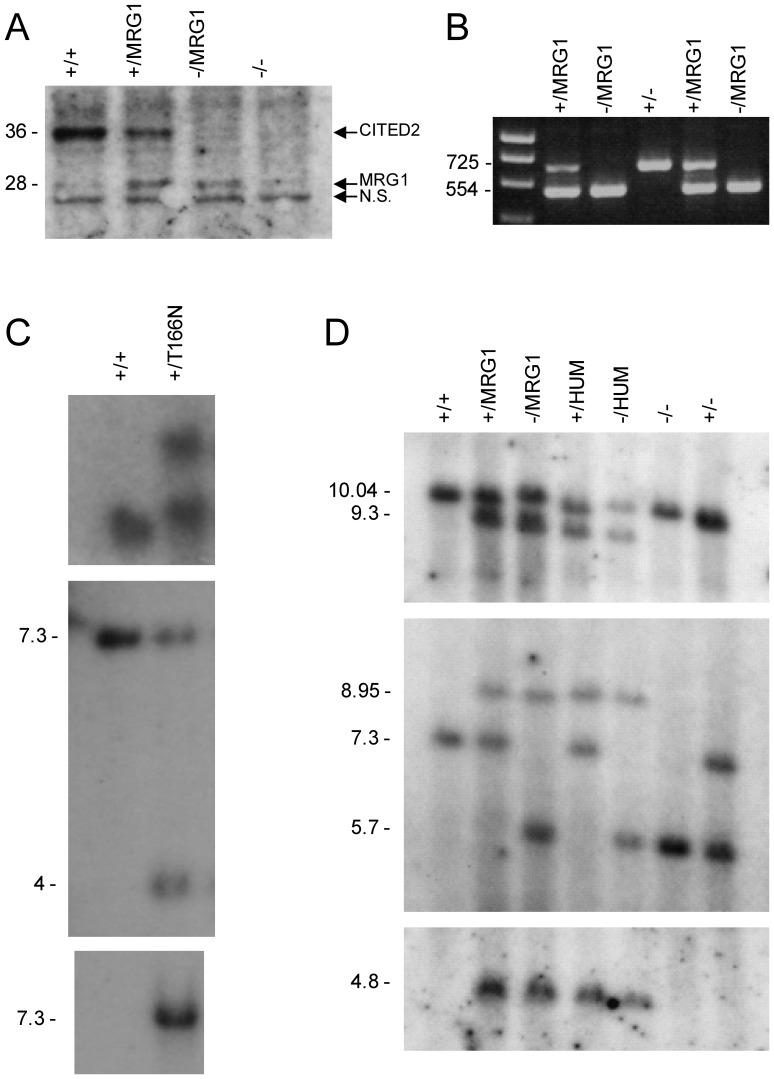
Molecular characterization of the *Cited2 ^T166N^*, *Cited2 ^MRG1^* and *Cited2 ^HUM^* alleles. (A) Western blot of total protein lysates from mouse embryonic fibroblasts (MEFs), probed with anti-CITED2 antibody. CITED2 and CITED2-MRG1 are indicated, as is a non-specific band (N.S.) that migrates at 25 kDa. (B) RT-PCR showing RNA products expressed by embryos of various genotypes. PCR primers were designed to differentiate between the endogenous mouse *Cited2* transcript and the *Cited2 ^MRG1^* transcript by their size difference. Wild type mouse *Cited2*, containing the SRJ domain produces the larger 725 bp band. (C) Southern blots of *Cited2 ^T166N^* allele. Top, Southern blot of *EcoRI* digested genomic DNA probed with a 5′-probe. Middle, Southern blot of *BglII* digested genomic DNA, probed with a 3′-probe. Probe positions are indicated in Fig. 3. Bottom, Southern blot of *SpeI* digested genomic DNA hybridized with an internal (Neomycin) probe, to confirm single copy integration. (D) Southern blots of *Cited2 ^MRG1^* and *Cited2 ^HUM^* alleles. Top, Southern blot of *EcoRI/SacII* digested genomic DNA, probed with a 5′-probe. Middle, Southern blot of *BglII* digested genomic DNA, probed with a 3′-probe. The position of the probes is indicated in Fig. 3. Bottom, Southern blot of *EcoRI* digested genomic DNA hybridized with an internal (Puromycin) probe to confirm single copy integration.

*Cited2 ^−/MRG1^* embryos, generated by crossing *Cited2 ^+/MRG1^* to *Cited2 ^+/−^* mice, were devoid of wild type *Cited2* and expressed only a single copy of the CITED2-MRG1 isoform. This was confirmed by western blotting and by Reverse Transcription Polymerase Chain Reaction (RT-PCR) followed by sequencing of the PCR products (Fig. 8A and B). 74 of 75 *Cited2 ^−/MRG1^* embryos harvested at 15.5 dpc and analyzed using MRI showed no overt cardiac or other anatomical developmental anomalies associated with loss of *Cited2*, and they all showed normal sized adrenal glands (Fig. 7D, I and N and Fig. S4). One embryo presented with ectopia cordis, small ventricular septal defect, oedema and other structural anomalies (Fig. S5). *Cited2 ^−/MRG1^* and *Cited2 ^MRG1/MRG1^* mice were found to be viable and fertile. Furthermore, *Cited2 ^−/MRG1^* mice were found at the expected Mendelian ratio at weaning (12/41, Table 2B, *X^2^ (3)*: 0.46, p = n.s.). The same analysis was carried out for the control, full length *Cited2 ^HUM^* allele. No anatomical anomalies were found in *Cited2 ^−/HUM^* embryos (0/20). *Cited2 ^HUM/HUM^* mice were also viable and fertile as were *Cited2 ^−/HUM^* (data not shown).

## Discussion

Our resequencing study reported here and those reported elsewhere [16], [17] indicate that non-synonymous mutations of *CITED2* can be observed in patients with congenital heart disease, and that these mutations tend to cluster in the SRJ domain. Cell-based assays investigating some of these variants have indicated that they can affect HIF1A-repression and/or TFAP2-coactivation functions of CITED2 [16]. The SRJ domain, although highly conserved in placental mammals, is substantially abbreviated in marsupials (e.g. the opossum *Monodelphys domesticus*) and in monotremes (e.g. the platypus *Ornithorhynchus anatinus*), and is absent in other vertebrates (Fig. 1B). Thus, the region may have appeared relatively recently in evolutionary terms and may conceivably be of relevance to differences in cardiac development and structure between placental mammals and other vertebrates [40]–[43].

Structurally, the SRJ region is predicted to be disordered and potentially functions as a flexible linker [24]. The T166 residue within this domain is predicted to be a target of proline directed kinases [25], [26], and our studies indicated that the T166 residue can be phosphorylated by MAPK1, and that activation of MAPK1 promoted co-activation function. Moreover cell-based studies indicated that the T166N mutation had a deleterious effect on TFAP2 co-activation function, and on the ability of CITED2 to promote ES cell proliferation in the absence of LIF.

Surprisingly, our *in vivo* study of mice carrying the T166N variant and the deletion of the SRJ domain indicate that in mice, under normal laboratory conditions, neither the complete deletion of the SRJ domain nor the introduction of a variant which could potentially lead to the loss of a phosphorylation site of CITED2 are detrimental to its function. In a single *Cited2 ^−/MRG1^* embryo out of 75 that we studied, we observed an ectopia cordis phenotype. Since ectopia cordis is not a phenotype which has previously been associated with the loss of *Cited2* [11], [15], [17], [21], [22], [44]–[47], and this embryo has normal adrenal glands (absence of which is a hallmark of *Cited2* deficiency), it is most unlikely that it is a consequence of the loss of the SRJ domain. Animals of this genotype are also viable and fertile. However, *in vitro* data indicate that the T166N mutation can have functional significance, and previous studies have indicated that other SRJ mutations also affect its function [16]. It is possible that the partial impairment of CITED2 function revealed by *in vitro* experiments is insufficient to affect development.

Taken together, the results obtained from mouse studies indicate that the SRJ domain is dispensable during mouse cardiac development and for viability and fertility. On the other hand, three independent human studies show that non-synonymous mutations, predominantly clustering in the SRJ domain, are mainly observed in patients with CHD and not in controls suggesting that this region is important for normal cardiac development. How can we explain these divergent observations? One possibility is that the SRJ is indeed dispensable for mammalian heart development, and that the observations from patients may be misleading. Supporting this idea, there is considerable lack of conservation in this domain between placental and non-placental mammals. Furthermore, the mutations in the SRJ do not significantly affect the disordered nature of the domain, indicating that it may be able to accommodate mutations without adversely affecting the overall structure and function of the protein. Moreover, in no case has it been shown that the mutation has either arisen *de novo* or been transmitted from an affected parent. Another possibility that partially reconciles the mouse and human observations is that variants found clustered in the SRJ in CHD patients may not, by themselves, be causative of disease and may require additional factors, such as second site genetic modifiers or environmental stress, for a phenotypic manifestation. Supporting this idea, we have previously shown that a maternal high-fat diet can alter the penetrance of left-right patterning defects and cleft palate in *Cited2* deficient mouse embryos [48]. In addition, we have also shown that *Cited2* can genetically interact with other developmental genes: loss of *Lmo4* can affect the *Cited2* phenotype [49]. A third possibility to be considered is human and mouse discordance where the mouse model fails to phenocopy the human disease. For instance, mutations in EVC and DHCR7 result in heart defects in humans (Ellis van Creveld Syndrome, OMIM 225500, Smith-Lemli-Opitz syndrome OMIM 270400) but not in the mouse [33], [50], [51].

To summarise, using a case-control approach we found, like others, that non-synonymous variants cluster in the SRJ region of CITED2 in CHD patients but not in controls. A point mutation (T166N) in this region greatly affects CITED2 co-activation function and LIF-independent growth of ES cells and is likely phosphorylated by MAPK1. We found that mice harboring, either the T166N point mutation or a deletion of the entire SRJ domain and 18 adjacent amino acids, undergo normal cardiac development and are viable and fertile. Thus, under normal conditions in mice, T166 and the SRJ domain are dispensable for these functions. We suggest that point mutations and deletions clustering in the SRJ region may require additional genetic or environmental factors to cause disease. Our results suggest that coding sequence mutations observed in case-control studies need validation using *in vivo* models and that predictions based on structural conservation and *in vitro* functional assays, or even *in vivo* global loss of function models, may be insufficient. This has implications for the interpretation of data arising from exon resequencing programs currently being pursued in cardiac and other developmental diseases.

## Materials and Methods

### Cases and controls

Patients (all of North West European White Caucasian ancestry) were ascertained through referring clinicians as part of the Genetic Origins of Congenital Heart Disease Study (GO-CHD) at several UK paediatric and adult congenital heart disease centres and were evaluated by physical examination and echocardiography. Samples were also obtained through collaboration with the University of Newcastle. Patients with CHD where the genetic mechanism was known were excluded (Down syndrome and DiGeorge syndrome amongst others). Ethnicity was determined by questionnaire and based on all four grandparents using the 2001 UK census of the population categories. Blood or saliva was collected from each affected individual and their parents, where available, and DNA was extracted using standard techniques. Written informed consent for inclusion in molecular genetic studies was obtained in each case. Approval for this study was obtained from a national ethics committee (MREC for Wales, REF 05/MRE09/89). 1227 unselected control samples were obtained from North West European White Caucasian individuals from the 1958 UK birth cohort (1041) and from the Wellcome Trust Case Control Consortium UK blood donor collection (186).

### DNA sequencing

Oligonucleotides were designed from flanking intronic and exonic sequence of the CITED2 gene (Accession No. AF129290) to produce two ∼500 bp PCR products. The following PCR primer pairs were used. To amplify the N terminal product: forward primer: 5′ TGTGGCGCGGGTCTCATTATC, reverse primer: 5′ CTGGTTTGTCCCGTTCATCTG; to amplify the C terminal product: forward primer 5′ TCACCCCTACCCCCACAACC, reverse primer 5′ TTCACGCCGAAGAAGTTGGG. Both strands of each product were sequenced on an automated ABI3730 sequencer using BigDye terminator cycle sequencing reagents (Applied Biosystems, CA, USA). Chromatographs were analyzed using PHREDPHRAP, Sequencher (v4.8, Gene Codes Corp., MI, USA) and GAP4 in house software (Wellcome Trust Sanger Institute, Cambridge, UK). Parental samples were analyzed where available to determine inheritance.

### Transient transfection and pull-down experiments

Plasmids, transfection conditions for transient transfection experiments, and luciferase assays have been previously described [10], [14]. Site directed mutagenesis was performed using the QuikChange™ Site-Directed Mutagenesis Kit (Stratagene) as per manufacturer's instructions, and oligonucleotides 5′-GCGGCGGCAGCAGCAACCCGGGCGGCTCGGGCGGC and 5′-GCCGCCCGAGCCGCCCGGGTTGCTGCTGCCGCCGC were used to create the T166N mutation which was confirmed by sequencing. GST pulldown experiments and desferrioxamine treatments were performed as previously described [10]. MAPK1 expression plasmid was a gift from Ralf Janknecht [52]. MAPKK1, MAPKK1S221A and MAPKK1-S217ES221E plasmids were gifts from Chris Marshall [53].

### Identification of phosphorylation sites in CITED2

Phosphorylation of CITED2 by active GST-ERK2 was carried out as described [54]. Wild type human GST-CITED2 (∼2 mM) or mutant GST-CITED2-T166N were incubated for 60 min at 30°C with activated human GST-ERK2 (2 U/ml), 10 mM magnesium acetate and 1 mM [γ^32^P]ATP (Amersham Bioscience) in 50 mM Tris-HCl, pH 7.5, 0.1 mM EGTA, 0.1 mM sodium orthovanadate and 0.1% (v/v) 2-mercaptoethanol. The procedure for mapping the phosphorylation sites is detailed elsewhere [55]. The ^32^P-labelled protein was reduced with DTT and incubated with 0.5% (v/v) iodoacetamide to alkylate cysteine residues and subjected to SDS-PAGE. The band corresponding to ^32^P-labeled GST-CITED2 was excised, digested with trypsin and chromatographed on a Vydac C18 column (218TP5215, 2 mm i.d. ×15 cm) equilibrated in 0.1% (v/v) trifluoroacetic acid (TFA). The column was developed with a gradient of acetonitrile in 0.1% (v/v) TFA from 0–30% acetonitrile (0–90 min), 30–50% acetonitrile (90–110 min) and 50–100% acetonitrile (110–120 min). The flow rate was 0.2 ml/min, fractions of 0.1 ml were collected and the ^32^P radioactivity determined by Cerenkov counting. Briefly, sites of phosphorylation within the peptides were determined by a combination of MALDI-TOF and MALDI TOF-TOF (matrix assisted laser-desorption ionization-time-of-flight/time-of-flight) mass spectrometry on an Applied Biosystems 4700 TOF/TOF Proteomics Analyser (utilising 5 mg/ml alpha cyano-cinnamic acid in 10 mM ammonium phosphate 50% acetonitrile as the matrix), and solid-phase Edman sequencing of peptides coupled to a Sequelon-arylamine membrane (on an Applied Biosystems 494C protein sequencer). The release of ^32^P radioactivity after each cycle of Edman degradation was counted. Phosphoaminoacid analysis was performed as described [54].

### ES cell pluripotency

E14/T cells [56] (gift from Austin Smith) were transfected with pPyCAGIP-*CITED2* or *CITED2-T166N* episomal expression vectors, pPyCAGIP-*Nanog* vector (positive control) and empty vector [36]. Cells were selected with puromycin, and then tested for maintenance of cell proliferation in the absence of LIF. Cell proliferation was assayed using crystal violet staining as described [13].

### Generation of *Cited2 ^T166N^* mice

The *T166N* mutation was introduced into the endogenous *Cited2* gene by gene targeting in murine 129Sv embryonic stem (ES) cells (Fig. 6A). The targeting vector was constructed using *Cited2* genomic sequences isolated from a λFIXII-129SvJ genomic library (Stratagene). The C-A change which converts Threonine 166 to Arginine was introduced via site directed mutagenesis which also introduced an adjacent, silent *PasI* restriction site 7 bp downstream. Further cloning and primer information is available upon request. The *frt-PGK-NeoR-frt* selection cassette was inserted 1.581 kb downstream of the stop codon. A DTA cassette was also introduced downstream of the 3′ homology arm for negative selection. The targeting vector was linearized with *NheI* and electroporated into ES cells at GenOway (France). ES cells were selected in 350 ug/ul G418. Correct targeting of the *Cited2* gene at the 3′arm was detected by long range PCR spanning the homology region and at the 5′arm by Southern blotting (data not shown). Correctly targeted ES cell clones were injected into C57BL/6J blastocysts and chimeras crossed to C57BL/6J females. Following successful germline transmission, targeting was confirmed to be correct by Southern blotting using standard protocols (Fig. 8C). Heterozygous mice were crossed to FLPeR [57] mice in a C57BL/6J background to remove the *frt-PGK-NeoR-frt* selection cassette. The resulting FLP recombined *Cited2 ^+/T166N^* mice were backcrossed to C57BL/6J for at least 2 further generations prior to the start of experiments and maintained by backcrossing to C57BL/6J mice. All studies involving animals were performed in accordance with UK Home Office Animals (Scientific Procedures) Act 1986 and approved by the University of Oxford's Local Ethical Review Process.

### Generation of the *Cited2^attP^* ES cell line

The *Cited2^attP^* ES cell line was generated via homologous recombination in C57BL/6N ES cells (Fig. 6B). A targeting construct was assembled by introducing two PhiC31 integrase *attP* sites into the *Cited2* locus of genomic DNA isolated from BAC RP23-450-B12 DNA. The 5′ *attP* site was introduced into the first intron, upstream of the ATG and the 3′ *attP* site 1.551 kb downstream of the stop codon as part of the Neomycin selection cassette, in between the PGK promoter and the Neomycin coding region. A DTA cassette was also introduced downstream of the 3′ homology arm for negative selection. Further details on the cloning are available upon request. The *NotI* linearized vector was electroporated into mouse JM8.F6 ES cells, a C57BL/6N ES cell line [58], followed by selection in G418 (175 µg/ml). Neomycin resistant colonies were analyzed by long range PCR spanning both the 3′ and 5′ homology arms for correct targeting of the *Cited2* gene and integration of the 5′ *attP* site. Primer pairs used for long range PCR were the following: for the 5′ homology arm: forward primer 5′ CCAGGATGGGAAACCCTGACT and reverse primer 5′ CTCAGTTGGGGGCGTAGTTCG, and for the 3′ homology arm: forward primer 5′ CCCTACCCGGTAGAAGTTCCT and reverse primer 5′ AGATATCACGTAGGATCTGCT. The generated ES cells were validated as being suitable for subsequence manipulation by injection into albino C57BL/6J (C57BL/6J-*Tyr^c-2J^*/J) blastocysts. Successful germline transmission was confirmed by the aforementioned long range PCR. Mice carrying the *Cited2 ^attP^* allele were bred to homozygosity and *in trans* to a *Cited2* null allele (*Cited2 ^tm1Bha^*) [11] to ensure that the tagged *Cited2* allele did not disrupt CITED2 function and to validate the ES cell resource for future manipulation.

### Generation of *Cited2 ^MRG1^* and *Cited2 ^HUM^* alleles by Recombinase Mediated Cassette Exchange (RMCE)

*Cited2 ^MRG1^* and *Cited2 ^HUM^* targeted ES cell lines were generated via RMCE using the previously targeted *Cited2 ^attP^* ES cells (Fig. 6C). Exchange vectors were designed to only replace the mouse *Cited2* ORF with either human full length *CITED2* or *CITED2^MRG1^* ORF, whilst retaining the intact mouse 5′ and 3′ UTRs. The vectors were assembled by introducing one *attB* PhiC31 integrase recognition site upstream of either human full length *CITED2* or *MRG1* at the same location in the first intron where the *attP* recognition site had previously been inserted. The second *attB* site was introduced upstream of the promoterless puromycin expression cassette. Successful RMCE was thus designed to reconstitute a functional puromycin resistance cassette.

1×10^6^ *Cited2 ^attP^* ES cells were co-electroporated with 5 µg of either the full length human *CITED2* or the *CITED2 ^MRG1^* exchange plasmid and 5 µg of pPhiC31o [59] using the Neon transfection system (Invitrogen) (3×1400 V, 10 ms) and plated on puromycin resistant fibroblast feeder layers. After approximately 7 days of selection in 600 ng/ml puromycin, 24 resistant colonies were isolated per construct, expanded and screened by PCR for the correct cassette exchange events at the 5′ and 3′ ends using specific primers which amplify across the the resulting *attL* and *attR* sites (to amplify across the *attL*: forward primer 5′ CAGCAGGTCCGCCGAGGTAGC and reverse primer 5′ AACCGAGACCGGTTCAACAGC, and to amplify across the *attR*: forward primer 5′ CACGCTTCAAAAGCGCACGTCTG and reverse primer 5′ CGCGTGAGGAAGAGTTCTTGCA). The generation of the *attL* site was confirmed by digesting the PCR product with SacII. Correct exchange was further confirmed by long range PCR (For 5′ homology arm: 5′ CCAGGATGGGAAACCCTGACT and 5′ CAGCAGGTCCGCCGAGGTAGC, and for the 3′ homology arm: 5′ CAGAAATCGCAAAGACGGAAG and 5′ AGATATCACGTAGGATCTGCT) followed by sequencing of the PCR products.

ES cells from correctly exchanged colonies were injected into albino C57BL/6J blastocysts. The resulting chimeras were mated with albino C57BL/6J females. Successful germline transmission yielded black pups and was confirmed by long range PCR and Southern blotting for correct targeting and exchange at both 5′ and 3′ ends. F1 pups were also screened by Southern blotting to ensure that no ectopic integration of the exchange cassette had occurred (Fig. 8D). Southern blotting was performed using standard protocols. Mouse colonies were maintained by backcrossing to C57BL/6J mice.

### Cell Culture

Mouse ES cells were cultured in Knockout DMEM (Dulbecco's Modified Eagle Medium, Invitrogen) supplemented with 2 mM L-Glutamine (PAA), 1× non-essential amino acids (PAA), 0.1 mM β-mercaptoethanol (Sigma), 1000 U/ml ESGRO (Millipore) and 10% fetal bovine serum (Invitrogen). Hep3B cells (ATCC No. HB-8064) were grown in Minimum Essential Medium (MEM) supplemented with 2 mM L-glutamine, 1% penicillin-streptomycin, and 10% FBS. All cells were cultured at 37°C in a humidified atmosphere containing 5% CO_2_.

### Generation and analysis of embryos

*Cited2 ^+/T166N^*, *Cited2 ^+/MRG1^* and *Cited2 ^+/HUM^* were crossed to mice heterozygous for the *Cited2 ^tm1Bha^* null allele (*Cited2 ^+/−^*). Embryos were dissected at 15.5 dpc and harvested and processed for Magnetic Resonance Imaging (MRI) as previously described [11]. MRI was performed on a horizontal 9.4 T/21 cm VNMRS Direct Drive™ MR system (Varian Inc., Palo Alto, CA, USA) as described previously [60]. Genotype deviation from expected Mendelian ratios was analysed using the *X^2^* test. The calculated probability of a type 1 error was calculated using the CHIDIST function in Excel (Microsoft Office 2007, Microsoft, Redmond, WA, USA).

### Western Blotting

Mouse embryonic fibroblasts (MEFs) isolated using standard protocols from decapitated and eviscerated 13.5 dpc embryos were lysed in lysis buffer (50 mM Tris, 150 mM NaCl, 0.5% Tx100) containing protease inhibitors (cOmplete, EDTA-free Protease Inhibitor Cocktail, Roche) at 4°C for 20 minutes, followed by centrifugation and removal of the non-soluble pellet. Protein concentrations were quantified using the Bicinchoninic acid (BCA) protein assay kit (Pierce). 60 ug of whole cell lysate were loaded into each well of a NuPage NOVEX 4–12% Bis-Tris gel (Invitrogen) and blotted onto a nitrocellulose membrane using standard protocols. Western blots using Hep3B cells were processed in a similar manner. Mouse monoclonal antibody against CITED2 (JA22) was used at 1∶500 (Abcam).

### RT-PCR

Total RNA was isolated from the caudal half of 13.5 dpc embryos using the RNeasy Tissue Kit (Qiagen) and first strand cDNA was synthesized using Quantitect Reverse Transcription Kit (Qiagen) as per manufacturer's instructions using 1 ug of total RNA. 1/20^th^ of the reverse transcription product was used per PCR reaction. PCR products were gel purified and sequenced to confirm identity. The following PCR primers were used: forward primer 5′ CAGAAATCGCAAAGACGGAAG and reverse primer 5′ CTCGTCGATGAAATCAGTGTC.

## Supporting Information

Figure S1 **Disorder plot of mouse wild type CITED2 and variants.** RONN (http://www.strubi.ox.ac.uk/RONN) was used to predict protein disorder. The highest peak, representing the highest probability of disorder, resides over residues corresponding to the SRJ (161–199) (red box). The CR2 domain (green box) has the lowest probability of disorder, consistent with it harboring all known biological functions of CITED2. CR1 (blue box) and CR3 (purple box) are also marked in the graph for the wild type protein. The location of the molecular lesions in each variant is indicated with a black arrow. The location of the SRJ and flanking residues which have been removed in the MRG1 isoform is marked with the red arrow.
(TIF)

Figure S2 **CITED2 T166N mutation does not impair CITED2's ability to repress HIF1 transactivation.** (A) Hep3B cells were transiently cotransfected with GAL4-HIF1A (40 ng), 3xGAL4-luciferase reporter (100 ng), CMV-lacZ (100 ng), and increasing amounts (4 and 40 ng) of CITED2 or mutant plasmids. GAL4-HIF1A transactivation was stimulated by adding desferrioxamine (DFO, 100 µM) as indicated. Results are presented as in (Fig. 2). (B) Western blots were performed using whole cell extracts prepared from Hep3B cells transfected with the indicated CITED2 plasmids. CITED2 was detected using a monoclonal anti-CITED2 antibody (top panels). Loading was monitored by probing the membrane with a monoclonal anti-β-tubulin antibody (bottom panels). (C, D) *Top panels:* Autoradiograms of gels showing the binding of 35S-labelled CITED2 and CITED2-p.T166N to GST (lanes 3, 4, 9, 10), GST-TFAP2A (lanes 5 and 6) and GST-p300CH1 (lanes11–12). *Bottom panels:* Coomassie blue stain of the gels showing relative amounts of GST, GST-TFAP2A and GST-EP300CH1 proteins. (E) Hep3B cells were transfected with CITED2 plasmids expressing the indicated CITED2 proteins. These were detected forty-eight hours after transfection by indirect immunofluorescence, using a monoclonal anti-CITED2 antibody and a secondary rabbit anti-mouse antibody coupled to FITC (green). Nuclei were counterstained with DAPI (blue). The merged image is shown in the panels on the extreme right.
(TIF)

Figure S3 **Co-expression of MAPK1 or MAPKK1 did not affect the expression of CITED2.** Western blot were performed using whole cell extracts prepared from Hep3B cells co-transfected with the plasmid expressing CITED2, a plasmid expressing MAPK1 and with plasmids expressing a kinase inactive MAPKK1 (SV40-MAPKK1-S221A) or a constitutively active MAPKK1 (SV40-MAPKK1-S217ES221).
(TIF)

Figure S4 **Adrenal gland size measurements.** The volume of adrenal glands of 15.5 dpc embryos were measured by segmentation analysis using Amira 5.3 (Visage, Berlin). All measurements were corrected for embryo weight. Values represent measurements obtained from 6 embryos from each genotype. Error bars represent SEM.
(TIF)

Figure S5 **E15.5** ***Cited2 ^−/MRG1^*** **embryo with ectopia cordis.** Single embryo out of 75 of the same genotype to present with any structural developmental anomaly. (A) External side view, (B) frontal view and (C) MRI image of sagittal section through embryo showing the ectopic heart (arrowhead) rostral to the umbilical hernia (arrow); the left forelimb has been removed. (D) Sagittal section through left adrenal gland (Ad). (E) Transverse section through the heart showing the heart outside the chest cavity and (F) 3D reconstruction of the heart and major vessels showing normal topology of the right and left ventricles (RV, LV) and a small ventricular septal defect (VSD). RA, Right Atria; LA, Left Atria; IVS, intraventricular septum; Tr, Trachea; AoA, Aortic Arch; DAo, Dorsal Aorta. Scale bars: 0.5 mm.
(TIF)

Text S1 (DOCX)

Table S1 **Diagnostic characteristics for all 1126 cases sequenced. Many patients have multiple diagnoses and for these the patient is recorded in the diagnostic category for the lesion most likely to need clinical intervention.** Abbreviations: Transposition of the great arteries (TGA; cc – congenitally corrected), secundum atrial septal defect (ASD), Tetralogy of Fallot (TOF), atrioventricular septal defect (AVSD), ventricular septal defect (VSD), pulmonary atresia with intact ventricular septum (PA/IVS), pulmonary atresia with ventricular septal defect (PA-VSD), coarctation of aorta (CoA), aortic stenosis (AS), hypoplastic left heart syndrome (HLHS), mitral valve abnormalities (MV abn), patent ductus arteriosus (PDA), double outlet right ventricle (DORV), pulmonary stenosis (PS), common arterial trunk (CAT), aorto-pulmonary (AP) window, tricuspid atresia (TA), double inlet left ventricle (DILV), discordant ventriculo-arterial connections (discordant VA) and partial and total anomalous venous drainage (PAPVD and TAPVD respectively).
(DOCX)
