# Supplementary figures and images for: Functional Significance of SRJ Domain Mutations in CITED2
Source: PLoS One. 2012 Oct 17;7(10):e46256. doi: 10.1371/journal.pone.0046256 (PMC3474824; doi:10.1371/journal.pone.0046256)

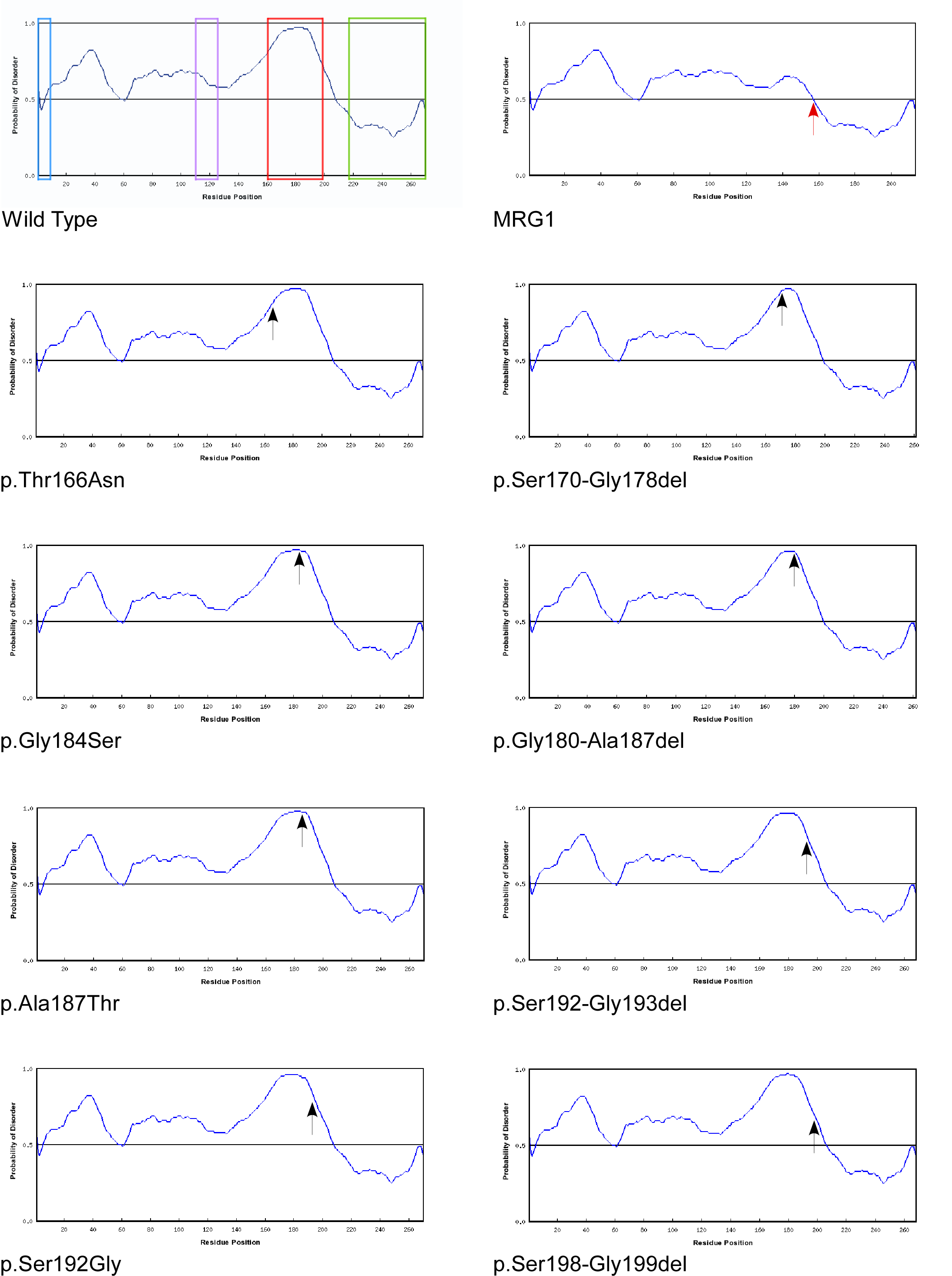

Supplement: Figure S1 — Disorder plot of mouse wild type CITED2 and variants. RONN (http://www.strubi.ox.ac.uk/RONN) was used to predict protein disorder. The highest peak, representing the highest probability of disorder, resides over residues corresponding to the SRJ (161–199) (red box). The CR2 domain (green box) has the lowest probability of disorder, consistent with it harboring all known biological functions of CITED2. CR1 (blue box) and CR3 (purple box) are also marked in the graph for the wild type protein. The location of the molecular lesions in each variant is indicated with a black arrow. The location of the SRJ and flanking residues which have been removed in the MRG1 isoform is marked with the red arrow. (TIF) [file pone.0046256.s001.tif]

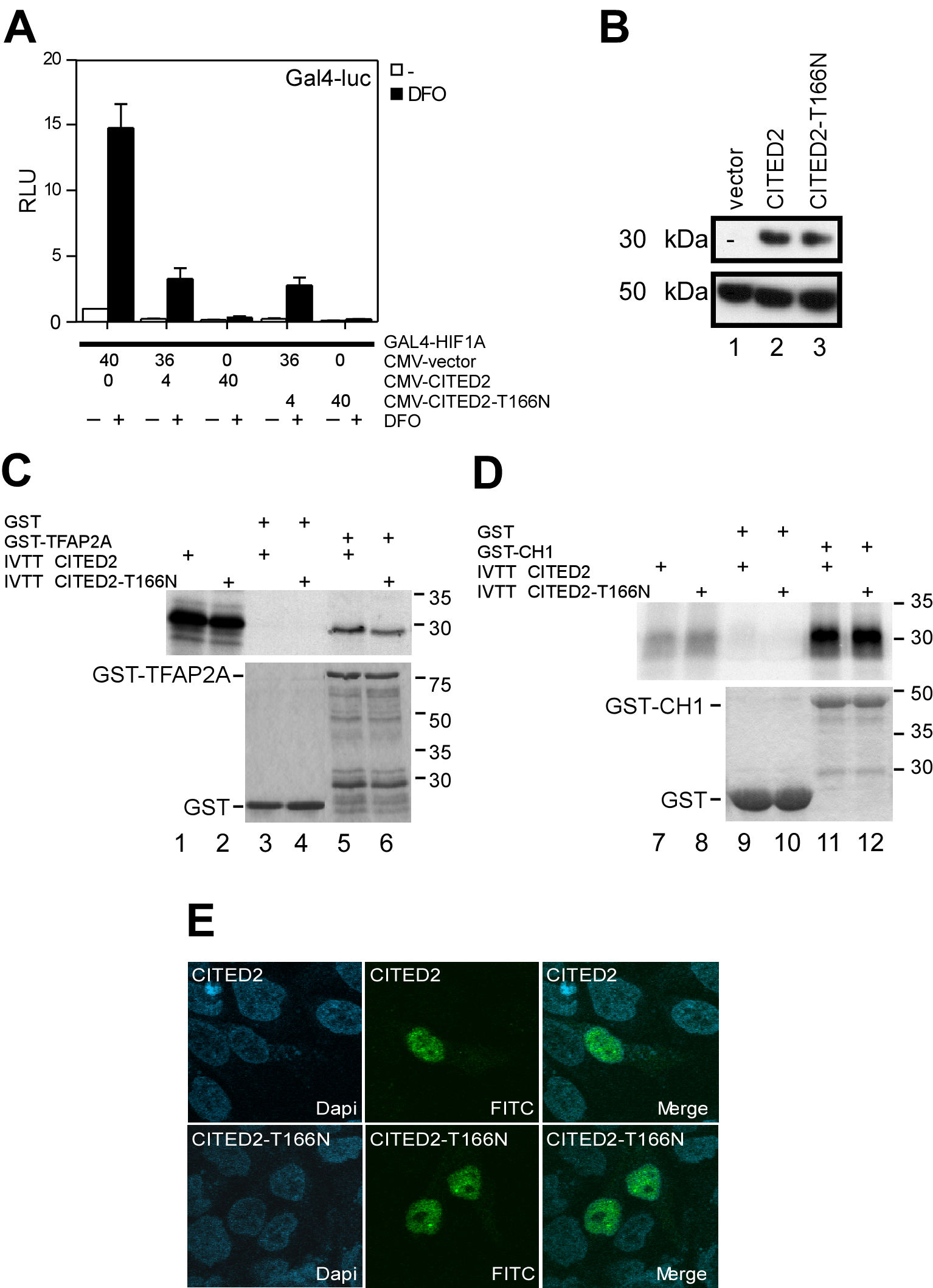

Supplement: Figure S2 — CITED2 T166N mutation does not impair CITED2's ability to repress HIF1 transactivation. (A) Hep3B cells were transiently cotransfected with GAL4-HIF1A (40 ng), 3xGAL4-luciferase reporter (100 ng), CMV-lacZ (100 ng), and increasing amounts (4 and 40 ng) of CITED2 or mutant plasmids. GAL4-HIF1A transactivation was stimulated by adding desferrioxamine (DFO, 100 µM) as indicated. Results are presented as in (Fig. 2). (B) Western blots were performed using whole cell extracts prepared from Hep3B cells transfected with the indicated CITED2 plasmids. CITED2 was detected using a monoclonal anti-CITED2 antibody (top panels). Loading was monitored by probing the membrane with a monoclonal anti-β-tubulin antibody (bottom panels). (C, D) Top panels: Autoradiograms of gels showing the binding of 35S-labelled CITED2 and CITED2-p.T166N to GST (lanes 3, 4, 9, 10), GST-TFAP2A (lanes 5 and 6) and GST-p300CH1 (lanes11–12). Bottom panels: Coomassie blue stain of the gels showing relative amounts of GST, GST-TFAP2A and GST-EP300CH1 proteins. (E) Hep3B cells were transfected with CITED2 plasmids expressing the indicated CITED2 proteins. These were detected forty-eight hours after transfection by indirect immunofluorescence, using a monoclonal anti-CITED2 antibody and a secondary rabbit anti-mouse antibody coupled to FITC (green). Nuclei were counterstained with DAPI (blue). The merged image is shown in the panels on the extreme right. (TIF) [file pone.0046256.s002.tif]

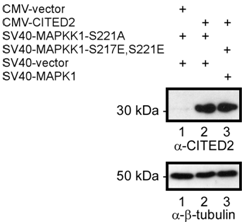

Supplement: Figure S3 — Co-expression of MAPK1 or MAPKK1 did not affect the expression of CITED2. Western blot were performed using whole cell extracts prepared from Hep3B cells co-transfected with the plasmid expressing CITED2, a plasmid expressing MAPK1 and with plasmids expressing a kinase inactive MAPKK1 (SV40-MAPKK1-S221A) or a constitutively active MAPKK1 (SV40-MAPKK1-S217ES221). (TIF) [file pone.0046256.s003.tif]

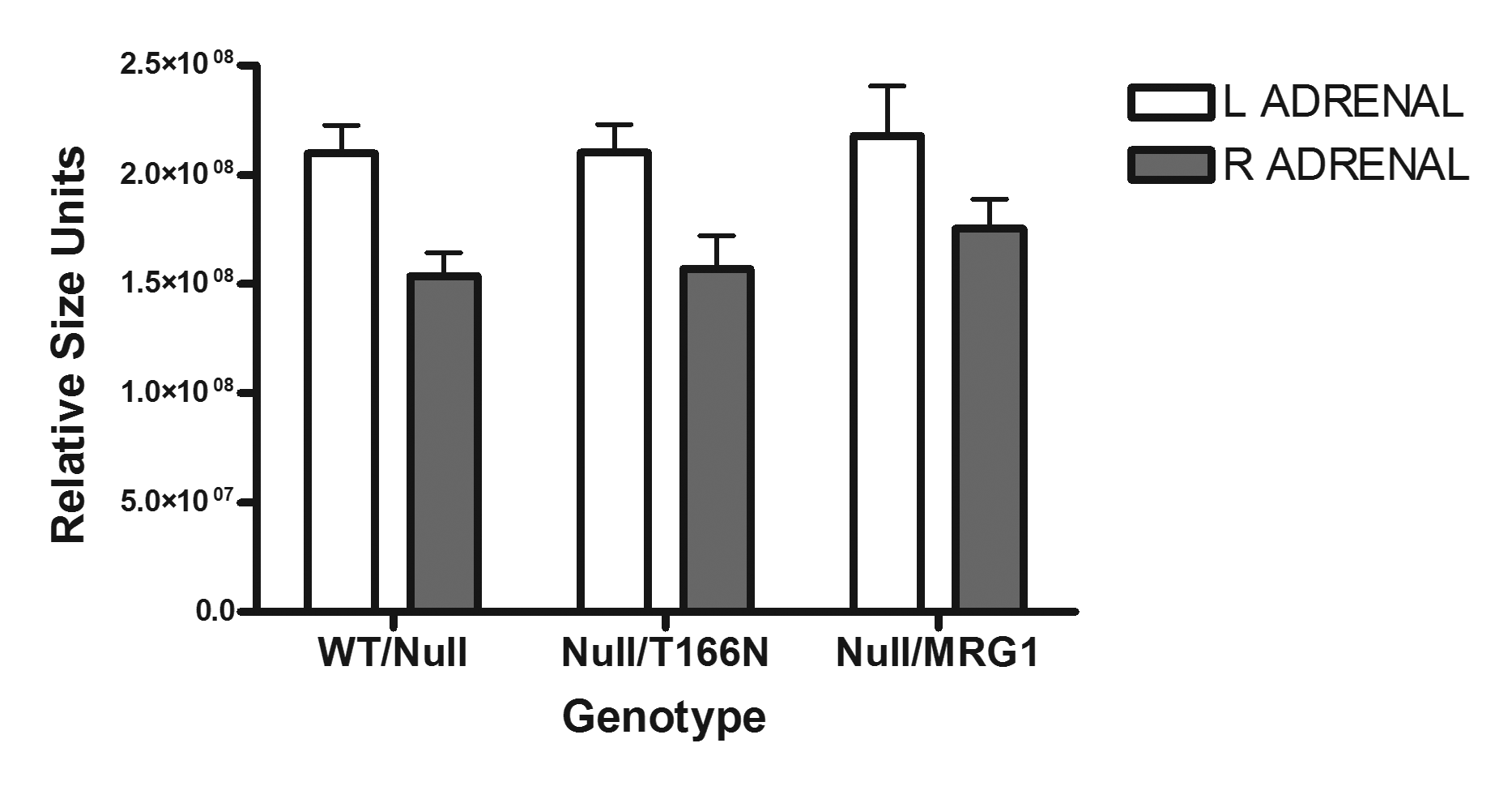

Supplement: Figure S4 — Adrenal gland size measurements. The volume of adrenal glands of 15.5 dpc embryos were measured by segmentation analysis using Amira 5.3 (Visage, Berlin). All measurements were corrected for embryo weight. Values represent measurements obtained from 6 embryos from each genotype. Error bars represent SEM. (TIF) [file pone.0046256.s004.tif]

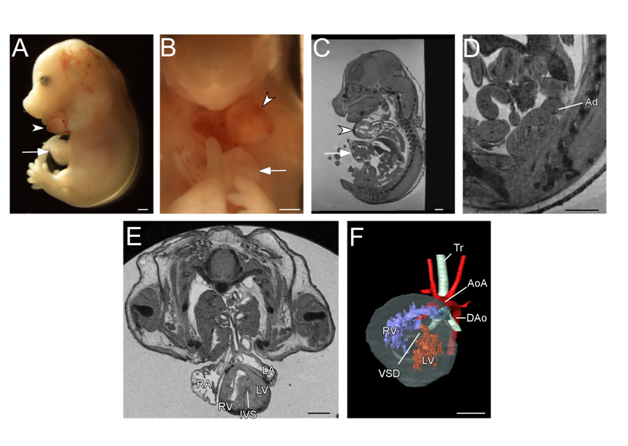

Supplement: Figure S5 — E15.5 Cited2 −/MRG1 embryo with ectopia cordis. Single embryo out of 75 of the same genotype to present with any structural developmental anomaly. (A) External side view, (B) frontal view and (C) MRI image of sagittal section through embryo showing the ectopic heart (arrowhead) rostral to the umbilical hernia (arrow); the left forelimb has been removed. (D) Sagittal section through left adrenal gland (Ad). (E) Transverse section through the heart showing the heart outside the chest cavity and (F) 3D reconstruction of the heart and major vessels showing normal topology of the right and left ventricles (RV, LV) and a small ventricular septal defect (VSD). RA, Right Atria; LA, Left Atria; IVS, intraventricular septum; Tr, Trachea; AoA, Aortic Arch; DAo, Dorsal Aorta. Scale bars: 0.5 mm. (TIF) [file pone.0046256.s005.tif]
