# Supplementary material for: Functional Significance of SRJ Domain Mutations in CITED2
Source: PLoS One. 2012 Oct 17;7(10):e46256. doi: 10.1371/journal.pone.0046256 (PMC3474824; doi:10.1371/journal.pone.0046256)
Supplement: Text S1 — (DOCX) [file pone.0046256.s006.docx]

***CITED2^His39del^***

Of the 5 variants found in both cases and controls (His39del, His160Leu, Ala7Ala, Gly194Gly and Gly194_Gly195del) only *CITED2^His39del^* was found to associate strongly with disease (10 of 1126 cases (0.89%) *vs.* 1 of 1227 controls (0.08%); χ^2^ p<0.005 (OR 10.94 {95% CI 1.40-85.5}). Eight of the ten cases with *CITED2^His39del^* had complex cardiac phenotypes including secundum ASD with dysmorphic features, pulmonary atresia ventricular septal defect (VSD), mitral atresia double outlet left ventricle, ASD with multiple VSDs, coarctation of aorta with bicuspid aortic valve, partial anomalous pulmonary venous return with ASD, congenital aortic stenosis, total anomalous pulmonary venous return and TOF.

To test whether association arose as a result of a genotyping error, genotyping of *CITED2^His39del^* was repeated in cases and controls. We also tested a replication cohort of 566 cases concurrently using the same assay along with a further set of 1248 UK Caucasian Controls from the Wellcome Trust Case Control Consortium UK Blood Donor collection. These were also not part of the original resequencing cohort. In all, 1524 cases and 2394 controls were successfully genotyped on a SEQUENOM® IPLEX platform (San Diego, USA). In total, 61 cases (4%) and 43 controls (1.8%) were found to carry the *CITED2^His39del^* variant (significant association χ^2^ p<5^e-5^ (OR 2.27 {1.03-3.39}). However, when results were analysed in their respective cohorts, original resequencing cohort *vs.* follow up replication cohort, the association is only identified in the original cohort with no significant association identified in the replication cohort (9/566 cases, 43/2394 controls ; χ^2^ p=0.92 (OR 0.89 {0.43-1.85}).

**Supplementary Materials and Methods**

**SEQUENOM assay**

Association of *CITED2^His39del^* variant with CHD was confirmed using a different genotyping platform using an iPLEX MALDI-TOF SEQUENOM assay (San Diego, USA). PCR and mass extension primers were designed using SEQUENOM’s assay design software (first primer 5’ ACGTTGGATGATTAGGGCGTTGAAGGCGTG 3’, second primer 5’ ACGTTGGATGCATGGGCATGGGGCAGTTC 3’, Tm 60°C). Multiplex PCR was performed in 10μl reactions with 1U Taq, 3.5mM MgCl2, 0.5mM dNTPs, 0.1μM of each primer and 20ng DNA. After PCR using standard conditions (95°C for 5 minutes followed by 35 cycles of 95°C for 30 seconds, 56°C for 30 seconds and 72°C for 1 minute) iPLEX extension reactions were performed according to manufacturer’s protocol.
