# Supplementary material for: Functional Significance of SRJ Domain Mutations in CITED2
Source: PLoS One. 2012 Oct 17;7(10):e46256. doi: 10.1371/journal.pone.0046256 (PMC3474824; doi:10.1371/journal.pone.0046256)
Supplement: Table S1 — Diagnostic characteristics for all 1126 cases sequenced. Many patients have multiple diagnoses and for these the patient is recorded in the diagnostic category for the lesion most likely to need clinical intervention. Abbreviations: Transposition of the great arteries (TGA; cc – congenitally corrected), secundum atrial septal defect (ASD), Tetralogy of Fallot (TOF), atrioventricular septal defect (AVSD), ventricular septal defect (VSD), pulmonary atresia with intact ventricular septum (PA/IVS), pulmonary atresia with ventricular septal defect (PA-VSD), coarctation of aorta (CoA), aortic stenosis (AS), hypoplastic left heart syndrome (HLHS), mitral valve abnormalities (MV abn), patent ductus arteriosus (PDA), double outlet right ventricle (DORV), pulmonary stenosis (PS), common arterial trunk (CAT), aorto-pulmonary (AP) window, tricuspid atresia (TA), double inlet left ventricle (DILV), discordant ventriculo-arterial connections (discordant VA) and partial and total anomalous venous drainage (PAPVD and TAPVD respectively). (DOCX) [file pone.0046256.s007.docx]

**Cardiac malformation n (%)**

Septal defects (ASD, VSD, PDA, AVSD) 266 (24)

Left heart lesions (AS, CoA, HLHS, MV abnormality) 127 (11)

Right heart lesions (TOF, DORV, PS, PA/IVS, PA-VSD) 556 (49)

TA, Ebstein malformation 29 (3)

DILV 14 (1)

CAT, AP window 5 (0.4)

TGA (+/-VSD), ccTGA 84 (7)

Complex isomerism, dextrocardia, situs inversus 29 (3)

PAPVD, TAPVD 8 (0.7)

Others 8 (0.7)
